# The Urgent Need for an Evidence-Based Digital Mental Health Practice Model of Care for Youth

**DOI:** 10.2196/48441

**Published:** 2024-03-22

**Authors:** Brad Ridout, Rowena Forsyth, Krestina L Amon, Pablo Navarro, Andrew J Campbell

**Affiliations:** 1 Cyberpsychology Research Group, Faculty of Medicine and Health, The University of Sydney, Sydney, NSW, Australia; 2 yourtown, Milton, QLD, Australia

**Keywords:** mental health, internet, digital health, telecounselling, social networking, telehealth, telemedicine, counseling, counselling, digital health, service, services, healthcare delivery, youth, model

## Abstract

Australian providers of mental health services and support for young people include private and public allied health providers, government initiatives (eg, headspace), nongovernment organizations (eg, Kids Helpline), general practitioners (GPs), and the hospital system. Over 20 years of research has established that many young people prefer to seek mental health support online; however, clear client pathways within and between online and offline mental health services are currently lacking. The authors propose a Digital Mental Health Practice model of care for youth to assist with digital mental health service mapping. The proposed model offers accessible pathways for a client to engage with digital mental health services, provides clear navigation to access support for individual needs, and facilitates a seamless connection with offline mental health services using a transferable electronic health records system. This future-looking model also includes emerging technologies, such as artificial intelligence and the metaverse, which must be accounted for as potential tools to be leveraged for digital therapies and support systems. The urgent need for a user-centered Digital Mental Health Practice model of care for youth in Australia is discussed, highlighting the shortcomings of traditional and existing online triage models evident during the COVID-19 pandemic, and the complex challenges that must be overcome, such as the integration of diverse mental health care providers and establishment of a robust electronic health records system. Potential benefits of such a model include reduced pressure on emergency rooms, improved identification of immediate needs, enhanced referral practices, and the establishment of a cost-efficient national digital mental health care model with global applicability. The authors conclude by stressing the consequences of inaction, warning that delays may lead to more complex challenges as new technologies emerge and exacerbate the long-term negative consequences of poor mental health management on the economic and biopsychosocial well-being of young Australians.

## Introduction

The COVID-19 pandemic highlighted what was already well-established about Australia’s mental health system: that demand and ease of access were not being successfully met [1-5], especially for individuals younger than 25 years, who are the most at-risk age group [6,7]. Over the past 20 years, the Australian youth mental health system has slowly transitioned from being fragmented and piecemeal, with poor integration between inpatient and outpatient services, to include online and offline offerings nationally [8]. However, these offerings are not always clearly connected for clients to navigate [1,7] and are often difficult to access in regional and remote areas (especially for young indigenous and marginalized populations of Australia), where online and offline services are seldom integrated or inclusive [9].

Young people are increasingly using the web as the first step in seeking support and information regarding their mental health [10,11]; yet, the digital services currently on offer often poorly integrate with existing offline mental health and hospital systems of care [1,12,13]. There is also a lack of sharing of clients’ mental health records between private and nongovernment organization (NGO) providers who offer online support (eg, ReachOut, BeyondBlue, Kids Helpline, and SANE), telephone centric services (eg, Lifeline and Kids Helpline), and traditional face-to-face clinical support (eg, headspace). This is due to several ethical and practical challenges associated with sharing electronic health records (EHRs) in mental health care [14], including issues of interoperability, missing data, privacy and confidentiality concerns, and legal compliance.

Currently, EHRs of young people are typically not part of digital mental health service offerings, with data often only collected upon entrance to determine the level of care required (ie, the triage stage). This means that when young people later return to online services at differing stages of need, or via a different modality, they often must “start again” with programs or offerings that may no longer suit their needs, rather than being directed to more suitable supports that reflect their prior engagement and the current stage of their mental health journey. Furthermore, the current lack of transferable EHRs between service providers implies that young people often have to retell their story multiple times. This can lead to incomplete patient histories, duplicate assessments, fragmented care, and safety risks associated with delayed or inappropriate treatment [15].

We propose a Digital Mental Health Practice model of care for youth (DMHP-Y) to aid digital mental health service mapping. It provides clear and accessible pathways for a young person to enter a digital mental health system, and then accurately navigate it to access support for their individual needs, beyond initial triage. This model proposes transferable EHRs regarding mental health service use to inform clinicians of historical online and offline service access, including any digital mental health assessments and treatments previously engaged in by a client. This will provide clinicians with essential background information on a patient’s mental health journey to date, assist in decision-making about future assessments and treatments, and facilitate continuity of care.

## A Brief Overview of Current Digital Mental Health Practices in Australia

Established in 2006, headspace is an NGO funded by the Australian Government as part of its national approach to service the mental health needs of young people (younger than 25 years) [16]. While headspace has been widely lauded for providing no-gap Medicare access to psychologists under a general practitioner (GP)–referred mental health care plan, the service has been under strain, given its high client demand nationwide [17,18] and the limitations to ongoing mental health management beyond the “Better Access” to mental health Medicare system [19-21]. “eheadspace,” the digital provision of mental health support, was established to provide online resources for young people to connect with mental health information and support through forums, digital tools, and one-to-one counseling via web chat and telephone. While not an emergency service, eheadspace duplicated some of the resources offered by other NGOs’ online services (eg, ReachOut and Kids Helpline), which have also struggled in connecting online resource offerings to offline clinician services (often because clients may choose to contact these services anonymously). Furthermore, the lack of integration between eheadspace and face-to-face headspace services demonstrates the challenges involved in integrating online and offline services, even within the same service environment. Herein lies a major systemic issue: the lack of secure, transferable, digital mental health recordkeeping within and between services [22-24].

Notwithstanding this challenge, new online platforms have emerged in recent years that leverage health ITs (HITs) to integrate digital mental health tools with existing face-to-face mental health services. The “Innowell” platform [25] is a customizable digital dashboard for mental health services, designed for assessing, monitoring, and managing the mental health of their clients. It collects and reports personal information to clients and their health professionals to promote collaborative and measurement-based care, and offers a range of online clinical content and assessment tools that are made available to clients as determined by their face-to-face mental health service. Another Australian example is Orygen’s “Moderated Online Social Therapy” (MOST) platform [26,27], a free online digital mental health service that young people can connect to following a referral from participating offline services. The MOST model of care is designed to support young people with, and in between, their face-to-face sessions with a clinician, or while they are waiting for care. It offers a range of on-demand digital services, including one-on-one support, self-directed online therapy, and moderated peer-to-peer online social networking.

These 2 platforms reflect emerging models of digital mental health care internationally, which also use HIT to augment face-to-face sessions and facilitate measurement-based care. A notable example from the United States is the “Digital Clinic” [28,29], which uses smartphone apps to augment and extend care at the Beth Israel Deaconess Medical Center in Boston, Massachusetts. This evolving hybrid model of care emphasizes therapeutic alliance, measurement-based care, and shared decision-making between clients and clinicians. A key feature of the model is mindLAMP (Learn, Assess, Manage, Prevent), an open-source app that provides customizable education, assessment, and management tools, and facilitates data sharing with patients and clinician support. mindLAMP can also be used to capture metadata regarding clients’ use of the app, as well as health and physical activity patterns via a smartwatch (eg, step count and heart rate) to enable “digital phenotyping.” Digital phenotyping is a new and evolving multidisciplinary field of science that uses data from smart devices to create a holistic digital picture of behavior and health, and has the potential to augment clinical care at the individual level (eg, by identifying clinically significant behavior changes) [30].

By integrating HIT with existing mental health services, the models described above represent the beginning of a new era of digital mental health service delivery. Something these models have in common is the requirement for clients to first connect with a face-to-face mental health service to access online services. However, young people are often reluctant to seek professional face-to-face help and face barriers to treatment such as cost, stigma, confidentiality concerns, poor mental health literacy, and inaccessibility to or lack of knowledge of resources [31,32]. These factors contribute to an increasing trend for young people to use the web as the first step in seeking support and information regarding their mental health [10,11], usually by typing a text-based query into an internet search engine [12]. Discussions of Rickwood et al’s [33] help seeking model in the context of the online environment emphasize the importance of internet-based resources and interventions in addressing the mental health concerns of young people [34] and ensuring that young people are guided to appropriate services [35]. Reputable digital mental health services that do not require prior face-to-face referrals, such as ReachOut and Kids Helpline, therefore play an important role in early intervention and as gateway services for young people in Australia who are seeking mental health information and support [34,36,37]. They also provide opportunities for young people to anonymously engage with peers online about mental health concerns, using moderated social media platforms, which itself has been shown to increase the likelihood of seeking formal mental health treatment [38,39]. A DMHP-Y is therefore required to map client-led online help seeking and engagement pathways to and within digital mental health services, and help clients navigate access to support for their individual needs, including seamless connections with offline mental health services.

## Considerations in Evidence-Basing a DMHP-Y

Digital mental health approaches are typically evidence-based as stand-alone interventions (eg, apps, video counseling, websites, and forums) rather than as holistic models of care. For a DMHP-Y to be effective and well-used, more research is needed to build an evidence base for the following, in order to inform design decisions that reflect youths’ help seeking behaviors and preferences, and the navigation of their mental health journey.

### Understand and Monitor Digital Engagement With Mental Health Care Over Time

This is necessary to map how and at what stages of their mental health journey young people engage with the digital mental health system over time, as use of digital mental health information and tools is often not linear. It also needs to be understood why some demographics might be more likely to arrive at a digital mental health service via an internet search, rather than via clinician referral or social networks. Further, in relation to online search words, search engine optimization (SEO) strategies must account for the different search terms individuals use to find information on mental health, given that the vocabulary for mental health help seeking has been shown to differ based on developmental age [40].

### Understanding Preferred Digital Tools of Young People for Supporting Their Mental Health

The online support services and modalities offered as part of a DMHP-Y must reflect and keep up with changing preferences and use patterns among young people. For example, there is growing evidence suggesting that young people want to use dynamic social media environments to seek information about their mental health and treatment options, and give and receive peer support from others facing similar mental health challenges [41-43]. While public social media platforms have significant privacy and security risks, recent studies have shown that custom-built social media–based services such as Kids Helpline’s “My Circle” (formerly “KHL Circles”) can be a safe and effective way for young people to engage in counselor-guided peer support for their mental health [25,44-46]. Next on the horizon is the increasing incorporation of virtual, augmented, and mixed reality technologies into social media, giving rise to the metaverse, which presents both opportunities and challenges for the future of digital mental health treatment and support [47,48]. A future-looking DMHP-Y model of care must therefore be dynamic enough to adapt quickly as technology and platform preferences continue to evolve, yet ensure that new service offerings are tested and evidence-based before implementation, and user privacy and safety remain the first priority.

### Ensuring an Optimum and Inclusive User Experience

It is well established that participatory design research methodologies involving all stakeholders and end users in the design and development of digital mental health systems and interventions are needed to ensure an optimum user experience for clients, clinicians, and other support staff [49,50]. However, further research into understanding individual human factors across different ages, genders, education, locations, abilities, neurotypes, and cultural needs is crucial to providing an inclusive and dynamic experience, leading to better service matching, more engagement, and less dropout from services. Furthermore, all DMHP-Y web content should be created using “universal design” principles to ensure usability by people with the widest possible range of abilities in the widest possible range of situations [51]. For example, Kids Helpline has already adopted a mobile-first approach and has implemented a range of inclusivity features to allow for people with hearing or sight impairment to access their services, such as “voice to text” across all modalities, ensuring that all website text is accessible via screen readers, and that all video content includes subtitles and audio cues. Kids Helpline has also introduced design initiatives to support children and young people with limited access to devices, data, and network coverage.

### Ensuring Continuity of Care

As discussed, a DMHP-Y model of care must provide for ongoing connections and transferability of information between online modalities and with offline services via EHRs. The need for and benefits of a transferable EHR should be made clear to clients upon first engagement with a digital mental health system. For example, so that they can easily navigate their mental health journey across modalities (ie, within and between online and offline services) and avoid having to repeat initial onboarding processes in the future. Participatory action research is needed to ensure that such a transferable EHR system is developed on the basis of user-centered design principles so that it meets the needs of both clinicians and clients, and effectively facilitates continuity of care (especially for nonlinear mental health treatment journeys). Once the evidence base for this is established, a standardized EHR system should ideally be implemented nationally across all community and private mental health services to ensure comprehensive and seamless record keeping between online and offline services.

My Health Records (Australia’s national digital health record system managed by the Australian Digital Health Agency) provides a case study in how difficult it can be to roll out an EHR system nationally. Challenges include low uptake, incomplete information, and concerns around privacy and security [52]. While it is possible for My Health Records to be used for mental health care, it is currently underused for this purpose, with concerns raised about the inclusion of sensitive and potentially stigmatizing mental health information alongside physical health information [24]. Furthermore, psychologists and other allied health professionals are excluded from engaging meaningfully with My Health Records, as conformant software is not currently available for allied health practices. My Health Records also does not allow for the inclusion of records of engagement with NGOs’ digital mental health services. Therefore, our perspective is that a DMHP-Y model of care requires a national EHR system specifically for mental health, which facilitates the transferring of records between digital mental health services and other health providers. While the considerations are complex and beyond the scope of this paper, any EHR system that seeks to support the mental health journeys of young people needs to include records of engagement with digital mental health services (most of which in Australia, at present, are NGOs and are outside of the health care system).

## Proposing a Future-Looking DMHP-Y

### Overview

**Figure 1.**
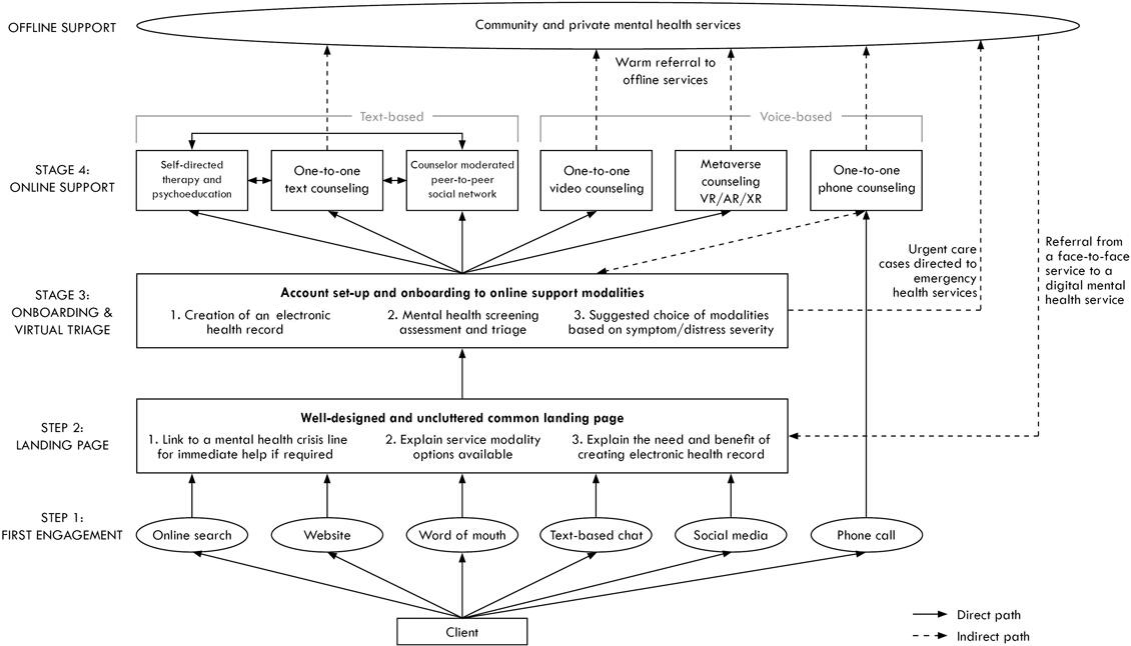
The future-looking Digital Mental Health Practice model of care for youth (version 1.0).

As outlined, current service provision for youth mental health in Australia offers both offline (eg, headspace and private practice allied health) and online support services (eg, eheadspace, Kids Helpline, and ReachOut). At the time of writing, the only emerging evidence-based digital platforms in Australia connecting clinicians and clients across online and offline service modalities are Innowell [25] and MOST [26,27]. While these models of care provide some basis for the model proposed below in Figure 1, they can only be accessed by clients after obtaining a referral from a participating offline mental health service.

In contrast, the DMHP-Y model of care proposed by the authors is focused on client-led online help seeking and engagement pathways. It aims to provide a map of the client-led engagement pathways that would be possible from the point at which a user first engages with a digital mental health service and is provided with a transferable EHR, regardless of whether they have previously engaged with offline mental health services or how far along their overall mental health journey they might be. While the model proposes that initial access to digital services (with the exception of phone counseling) should be via a common landing point for onboarding, it accounts for there being numerous engagement and re-engagement points (such as following engagement with offline services). The model also accounts for technologies that will change the way we interact online over the coming years, such as the incorporation of natural language processing and other artificial intelligence (AI) modalities, and avatar and social network use in the metaverse. The model presented is, therefore, a future-looking starting point for a model of care that would improve connections between services and transferability of client information, as technological advances and user preferences continue to evolve.

The proposed DMHP-Y model aims for what is herein dubbed the 3 Is of digital mental health care: *inclusivity* (equal access to all), *individuality* (choice based on preferences), and *integrity* (reliable, ethical, and evidence-based care). The 3 Is are addressed, where applicable, across each step of a client’s movement through the system of care.

### Step 1: First Engagement

This step represents the *individual* journeys and modalities that lead young people to first engage with a digital mental health service. As stated previously, young people often use the web as their first step in seeking support and information regarding their mental health [10,11], usually via a text-based query in an internet search engine, which is the most common help seeking approach among young people aged <25 years [12]. Other potential initial engagement channels include suggested links on websites or social media, recommendations from text-based chat services and social media, as well as clinician referral and word of mouth.

Research on young people’s mental health–related search word practices suggests that digital mental health services need to ensure that they can be easily found not only using diagnostic search terms (eg, depression, anxiety, etc), but also using simple affective phrases (eg, sad, scared, or lonely) [40]. SEO strategies also need to account for differences in demographics, ability, neurotype, language, and cultural backgrounds to ensure *inclusivity*. Moreover, ongoing analysis of common search phrases for mental health issues to inform SEO updates is key to a DMHP-Y model remaining a primary online source of help (*integrity*) over less evidence-based or predatory mental health offerings.

### Step 2: Landing Page

No matter what leads a potential client to first engage with a DMHP-Y system, they should be directed to a common landing page that quickly explains the service modality options available to them (*individuality*), as well as a clearly identifiable link to “immediate help” (ie, a link to a national mental health crisis line [53]) to reduce cognitive load on what could be a distressed individual (*integrity*). Often, landing pages are cluttered with dense information or vague navigation. Studies clearly show that time spent engaging with a digital platform is directly related to intuitive design and consideration of user digital literacy [54,55]. The design of the landing page is therefore crucial and should not be dictated solely by the service provider but rather be designed in consultation with young people from a diverse range of backgrounds and with differing levels of mental health literacy (*inclusivity*). This is to ensure that the right balance is achieved between the need to provide essential information to the client and the need of the client to progress to an appropriate service quickly and easily. This essential information should include explaining the need for and benefit of creating a transferable EHR to navigate their mental health journey, as the next step in the model may require this for progress into the service.

### Step 3: Onboarding and Virtual Triage

The next step is to create a transferable EHR for the client (if they do not already have one) so they can onboard into the DMHP-Y system and complete a mental health screening assessment to inform “virtual triage.” It has been proposed that virtual triage could also leverage AI, specifically natural language processing, which shows promise for adaptation for mental health services [55-58] and is currently being used successfully in corporate operations and retail services. It is not the authors’ suggestion that such a sensitive system and service model be reliant on AI alone, rather, a hybrid model of triage engagement be offered (ie, human and AI), depending on the level of severity of a client upon initial screening. An example of this would be when a client has completed the initial onboarding for an EHR and their mental health screen indicates high levels of distress (addressing *individual* urgent need), they are flagged as such and directed immediately to a human counselor contact (eg, text-based or voice-based, depending on the preference of the client) as the first port of call for immediate triage (ie, *integrity* of priority care). For clients who decline contact with a human counselor, the system may provide them with targeted resources and alternate options for support (*inclusivity*), while flagging their profile within the ecosystem for therapeutic monitoring, follow-up, and support. Clients identified as “severe” at screening can, if required, be directed to emergency services, or referred to community or private mental health services via a contact number or URL for their closest service.

### Step 4: Online Support

Once level of care requirements are established, the DMHP-Y model can offer a personalized (ie, *individual* and *inclusive*) menu of text- and voice-based choices from a suite of digital therapies and supports. Low-risk first-time clients may be directed to multimedia psychoeducation, or virtual counselor sessions can be scheduled in an environment of choice (eg, an initial telecounseling session via phone, web, or metaverse avatar counseling). This initial entrance level of support could also include evidence-based AI chatbots for mental health information [56,58] or asynchronous text-based guidance from counselors [59]. In turn, this would aid them to learn more about their mental health needs and options for individual care, both within the online service and via offline services. With appropriate technology and client consent in place, there is also potential for digital phenotyping to play a role in informing clinical support needs, by integrating data from a client’s smart devices with their engagement trends with the DMHP-Y model over time [30].

For clients who understand their mental health needs (ie, have previously received diagnosis or therapies), the option of self-guided psychoeducation and digital programs, such as cognitive behavioral therapy or acceptance and commitment therapy, may be offered. These forms of digital therapies are evidence-based for clients experiencing depression, anxiety, and stress symptoms and wanting to self-manage [60,61].

Clients who are seeking continuous social support, especially for chronic mental health conditions, may benefit from community engagement via counselor-moderated 24/7 peer-to-peer social networks. This level of ongoing social support has been found to benefit young people in managing their mood and learning new coping strategies, and offers social support outside of specific therapies [26,44-46]. Connecting for peer-to-peer support in counselor-moderated social networks such as Kids Helpline’s My Circle has also been shown to reduce mental health stigma and increase help seeking for mental health issues in the future [44-46]. Kids Helpline’s My Circle is an example of how peer-to-peer social networks can integrate and link directly to other text-based resources such as self-directed online psychoeducation modules, website content, and text-based one-to-one counseling in an easy-to-navigate digital ecosystem. The integration of digital mental health resources and interventions into an ecosystem further allows them to be used strategically and dynamically by digital counselors, by conceptualizing them on dichotomies from low to high intensity (eg, website vs one-to-one text-based counseling) or solo to assisted interventions. For example, clients presenting with “severe” symptomatology who are struggling to see improvements by using self-directed online psychoeducation may conceptually benefit from using higher-intensity and assisted interventions, which may involve online counselors directing them to peer-to-peer support or one-to-one interventions that match their willingness, motivation, and clinical needs.

### Offline Support

Though not a “step” in the DMHP-Y model, referral to offline face-to-face support may be deemed appropriate or necessary at any stage of a client’s engagement with a digital mental health service. In this situation, digital counselors should provide “warm referrals” for clients; that is, they should discuss options and gain client consent to introduce them to an offline service. A system for sharing of EHRs must therefore be established between a DMHP-Y model and offline services for seamless recordkeeping and best-practice care for clients in need of outpatient or inpatient care. This, of course, goes both ways, as clients may transition to digital services having already engaged with offline services. An EHR system for mental health should therefore ideally be implemented nationally across all community, private mental health, and digital mental health services and allow clients who may have an existing EHR established with offline services to connect it to digital mental health services (as many seek digital services themselves rather than via a referral from an offline service).

At the time of writing, there are multiple health record software programs on offer in Australia, but none provide seamless recordkeeping between online and offline services. There are, however, digital mental health services in Australia that seek to connect young people to and from offline services via direct referrals, albeit without fully transferable EHR systems. For example, Kids Helpline connects young people to offline services via warm referrals to community and specialist mental health services and emergency and child protection services where clients are at risk of significant harm. Conversely, as discussed, following a referral from participating offline services, young people can connect to Orygen’s MOST platform [26,27] to access online support either while they are waiting for face-to-face care in order to augment their face-to-face sessions or after discharge. It is these types of systems of care that need to be further developed and tested at the national level.

## Conclusion

Australia presently overrelies on traditional triage models of care for mental health (eg, hospital emergency departments and crisis support telephone services), which came close to systemic failure during the peak of the COVID-19 pandemic as they struggled to address increased demand for mental health support during the health crisis [62]. Given that the demand for online services is growing despite many digital mental health services currently having a poor evidence base [63], it is now even more necessary to develop a road map, testing phase, and rollout plan for a DMHP-Y model of care for service provision.

User-preferred models for mental health that provide EHRs to enable seamless cross-referrals between online and offline allied health and psychiatric care will reduce pressures on existing mental health providers in Australia and facilitate continuity of care for clients. This will be achieved by maintaining detailed records of the young person’s mental health journey—tracking engagement with both clinical treatment and digital support tools—which will allow seamless repeat entrance to both online and offline support when needed. The goal should be for young people to be able to enter services for support without having to retell their story wherever possible.

The primary challenge is the “start up” phase for a DMHP-Y model of care, which must address four significant hurdles: (1) synchronization of existing systems and models of mental health care, online and offline, across different provider organizations (NGOs, government, private sectors, GPs, and hospitals); (2) establishing a transparent, secure, and agile EHR system for mental health; (3) tracking and managing clients that present within digital systems under multiple identities (eg, due to stigma or privacy concerns); and (4) upskilling mental health workers and professionals in digital health and digital therapy skills to extend evidence-based practices across existing (eg, videoconference counseling) and future technologies (eg, metaverse counseling and AI). Developing a dynamic and future-looking DMHP-Y model of care that incorporates the introduction of new automated technologies such as AI does not imply the loss of quality mental health professional care. On the contrary, a DMHP-Y model of care that incorporates AI facilitates the economic and professional release of clinicians to dedicate time and expertise to clients who are in urgent need of help or require long-term care across hybrid offerings of traditional community face-to-face resources and digital resources.

The investment and development in DMHP-Y models of care will improve services in Australia from “treatment only” to “treatment and user-choice support” systems. In turn, this will (1) alleviate pressure on emergency rooms (which have seen a dramatic increase in mental health presentations over the past decade) [64]; (2) improve online to offline referral practices (and vice versa); (3) better identify and prioritize those who need immediate face-to-face help from those who need ongoing social and psychoeducation support; (4) greatly improve the cost efficiency of the current mental health system in Australia; and (5) pioneer a national model of digital mental health care that is replicable globally.

The most significant risk to our mental health system is inaction. If we do not start implementing changes in digital systems in mental health care at the national level, the knock-on effects of poor mental health care management for young people will continue into adulthood. This will contribute to significant long-term economic and biopsychosocial problems for Australia’s population in the future, compounding national health burdens of disability and disease. Moreover, the longer the delay to develop, test, and refine such digital models for mental health services, the more complex the task will become as new technologies and platforms such as AI and the metaverse are introduced at an increasingly rapid pace.

## Abbreviations

AI: artificial intelligence
DMHP-Y: Digital Mental Health Practice model of care for youth
EHR: electronic health record
GP: general practitioner
HIT: health IT
mindLAMP: Learn, Assess, Manage, Prevent
MOST: Moderated Online Social Therapy
NGO: nongovernment organization
SEO: search engine optimization
